# Double burden of malnutrition among women and children in Zimbabwe: a pooled logistic regression and Oaxaca-Blinder decomposition analysis

**DOI:** 10.3389/fpubh.2024.1451898

**Published:** 2024-09-12

**Authors:** Akim Tafadzwa Lukwa, Plaxcedes Chiwire, Folahanmi Tomiwa Akinsolu, Denis Okova, Charles Hongoro

**Affiliations:** ^1^Health Economics Unit, School of Public Health and Family Medicine, Faculty of Health Sciences, University of Cape Town, Cape Town, South Africa; ^2^Western Cape Department of Health, Cape Town, Western Cape Province, South Africa; ^3^Department of Health Services Research, CAPHRI Care and Public Health Research Institute, Maastricht University, Maastricht, Netherlands; ^4^Centre for Reproduction and Population Health Studies (CeRPHS), Nigerian Institute of Medical Research, Lagos, Nigeria; ^5^Developmental, Capable and Ethical State, Human Sciences Research Council, Pretoria, Gauteng Province, South Africa; ^6^School of Health Systems and Public Health (SHSPH), Faculty of Health Sciences, University of Pretoria, Pretoria, Gauteng Province, South Africa

**Keywords:** double burden of malnutrition (DBM), pooled logistic regression, Oaxaca-Blinder decomposition, nutritional outcomes, urban–rural disparities

## Abstract

**Background:**

The double burden of malnutrition (DBM) is a public health issue characterised by the coexistence of undernutrition and overnutrition within the same population, household, or individual. Undernutrition, manifesting as stunting, wasting, or being underweight, results from insufficient nutrient intake while overnutrition, manifesting as overweight or obesity, results from excessive caloric intake, poor diet quality, and sedentary lifestyles. This dual burden poses significant challenges for health systems due to lost productivity and increased healthcare expenditure.

**Methods:**

This study utilised data from the Demographic and Health Surveys (DHS) conducted in Zimbabwe for 2010–2011 and 2015, which provided information on women’s and children’s health and nutritional status, household characteristics, and socio-economic status. Pooled logistic regression was used to analyse the association between various sociodemographic factors and DBM among women and children. The Oaxaca-Blinder decomposition method explored differences in DBM between 2010–2011 and 2015.

**Results:**

The average age of mothers was approximately 31 years, and children’s ages averaged around 32 months. From 2010 to 2015, there was a notable socio-economic improvement, with a decrease in the percentage of mothers in the poorest quartile from 20 to 16% and an increase in the richest quartile from 22 to 23%. The study found a slight decrease in overall household DBM among women from 34% in 2010 to 32% in 2015, while DBM among children increased from 12 to 14%. Pooled logistic regression analysis indicated that children in rural areas had statistically significantly higher odds of experiencing DBM than their urban counterparts. The Oaxaca-Blinder decomposition showed that changes in residence status significantly impacted the increase in DBM among children. At the same time, the coefficient effect accounted for most of the unexplained differences in DBM among women.

**Conclusion:**

The growing DBM among women and children in Zimbabwe is significantly influenced by changes in residence status. The findings highlight the need for targeted public health interventions to address urban–rural disparities and emphasise the importance of considering socio-economic, environmental, and behavioural factors. Context-specific public health strategies, aligned with WHO’s Double Duty Actions, are essential to improve the nutritional health of Zimbabwe’s population.

## 1 Introduction

The double burden of malnutrition (DBM) is a significant public health issue encompassing the simultaneous presence of undernutrition and overnutrition within the same population, household, or individual (1). Undernutrition manifests as stunting, wasting, or being underweight, often due to insufficient nutrient intake or repeated infections (2). Conversely, overnutrition presents as overweight or obesity, typically resulting from excessive caloric intake, poor diet quality, and sedentary lifestyles (3–5). The coexistence of these forms of malnutrition poses unique challenges for health systems, as they must simultaneously address ends of the nutritional spectrum. DBM is associated with an increased risk of chronic diseases, impaired cognitive and physical development in children (6), higher morbidity and mortality rates (7), and substantial economic costs due to lost productivity and increased healthcare expenditure (8–10).

Globally, the prevalence of DBM is rising, driven by rapid urbanisation, economic growth, and globalisation of food markets (11). These factors contribute to changes in dietary patterns, with an increasing consumption of energy-dense, nutrient-poor foods and a decline in physical activity. According to the World Health Organization, over 2 billion people suffer from micronutrient deficiencies, while more than 1.9 billion adults are overweight or obese, and 462 million are underweight (12). The situation is particularly critical in Sub-Saharan Africa, where the traditional issues of food insecurity and undernutrition are now compounded by rising rates of overweight and obesity (13). In developing countries, including those in Sub-Saharan Africa, DBM is influenced by complex socio-economic transitions (14–17). Rapid urbanisation often leads to lifestyle changes favouring sedentary behaviour and unhealthy eating habits, contributing to overnutrition. Simultaneously, undernutrition persists due to ongoing issues such as poverty, food insecurity, and inadequate healthcare infrastructure. These dynamics are especially pronounced in low-income settings like Zimbabwe (18–20). The country faces significant economic challenges that impact food availability and accessibility (21), while urbanisation drives dietary shifts towards processed and fast foods high in sugars, fats, and salt. Consequently, Zimbabwe exemplifies the dual challenges of combating undernutrition and overnutrition (22), reflecting broader trends observed in many developing nations.

Understanding the prevalence and drivers of DBM in Zimbabwe is crucial for developing effective public health strategies. Addressing this dual burden requires a multifaceted approach considering the socio-economic and environmental contexts influencing dietary behaviours and health outcomes. By identifying and targeting the specific factors contributing to undernutrition and overnutrition, policymakers can design interventions that promote holistic nutritional health and mitigate the impacts of DBM on the population. The double burden of malnutrition (DBM) carries profound health consequences, manifesting in increased morbidity and mortality rates, particularly in vulnerable populations such as children and women of childbearing age (23). For children, undernutrition can lead to stunting, wasting, and underweight, all of which are associated with impaired cognitive and physical development (24, 25). Stunted children often face lifelong consequences, including reduced educational attainment and decreased economic productivity in adulthood (26). Furthermore, undernutrition weakens the immune system, increasing susceptibility to infections and diseases, which can lead to higher mortality rates among children (27).

The coexistence of undernutrition and overnutrition within the same population exacerbates the health burden, creating a complex scenario where individuals might suffer from deficiencies in essential nutrients while simultaneously dealing with excess calorie intake. For women, particularly those of childbearing age, DBM poses additional risks. Undernourished mothers are more likely to give birth to low birth-weight infants, perpetuating a cycle of malnutrition (28). On the other hand, overweight and obese women face higher risks of pregnancy complications, including gestational diabetes, pre-eclampsia, and complications during delivery. These health issues affect the mothers and have long-term implications for their children’s health and development. The socio-economic impact of DBM is substantial, affecting economic productivity, healthcare costs, and overall socio-economic development (29). Malnutrition, in various forms, reduces individual productivity by impairing physical and cognitive abilities. Stunted and underweight children are less likely to perform well academically, which limits their future employment opportunities and earning potential (30). Over time, this translates into a less skilled workforce, which hampers economic growth and development. Furthermore, the healthcare costs associated with DBM are significant. Treating conditions related to undernutrition, such as infections and growth monitoring, alongside managing NCDs associated with overnutrition, places a substantial financial burden on healthcare systems. This dual strain can divert resources from other essential health services, exacerbating a country’s overall health challenges. In low-income settings like Zimbabwe, where healthcare resources are already limited, the economic strain caused by DBM can be particularly detrimental, leading to an overstretched healthcare system that struggles to meet the needs of its population.

Additionally, DBM affects overall socio-economic development by perpetuating cycles of poverty and inequality (31). Addressing DBM is therefore crucial not only for improving health outcomes but also for promoting sustainable socio-economic development. Effective interventions can break the cycle of poverty and malnutrition, leading to healthier, more productive populations and fostering economic growth and development. This study’s importance lies in its ability to highlight the critical health and socio-economic consequences of DBM. By providing a detailed analysis of the factors contributing to DBM and identifying potential intervention points, the study aims to inform policy decisions and public health strategies that can mitigate the dual burden of malnutrition. This is essential for improving the health and well-being of populations, particularly in low-income settings like Zimbabwe, and promoting sustainable socio-economic development.

## 2 Methods

### 2.1 Data sources

Data for this study were obtained from two separate cross-sectional surveys, specifically the Demographic and Health Surveys (DHS) conducted in Zimbabwe for the years (32) and 2015 (33). These surveys are nationally representative and are designed to collect data from different participants during each survey cycle. Although the surveys were conducted in similar geographical areas to maintain comparability, the participants were not the same individuals across the two surveys. This cross-sectional design enables the assessment of trends in the double burden of malnutrition over time but does not involve tracking the same individuals longitudinally. These data sets provide information on women’s and children’s health and nutritional status, household characteristics, and socio-economic status (SES). The sample design for surveys was structured to ensure representativeness across various administrative levels, including national, urban, and rural areas, and each of Zimbabwe’s 10 provinces (32, 33). The 2015 ZDHS used the 2012 Population Census as its sampling frame and employed a stratified, two-stage cluster sampling design (33). In the first stage, 400 Enumeration Areas (EAs) were selected (166 urban and 234 rural). In the second stage, a complete listing of households within these EAs was conducted in March 2015, excluding institutional living arrangements (33). The 2010–2011 ZDHS followed a similar methodology, ensuring consistency and comparability between survey rounds. This study population consisted of women of childbearing age (15–49 years) and children from the selected households. The sample sizes were 7,387 women and 4,369 children in 2010–2011 and 7,965 women and 4,778 children in 2015.

### 2.2 Data preparation and variable definitions

Initially, the women’s dataset was merged with the children’s dataset using *caseid* as the identifier. Subsequently, the combined women and children dataset was merged with the household dataset. This integration allowed for an analysis of the double burden of malnutrition among women and children within the same households. Key variables were defined and generated for analysis. For children, the variable *Overweight status* was created based on the *wasting* variable, with values indicating overweight [1(>2 SD): Overweight] or not overweight [2 (<2 SD): Not overweight]. Other important sociodemographic variables included in the analysis were the *child’s age*, *mother’s education level*, *socioeconomic status* (measured using a wealth index categorised into five quintiles from poorest to richest), *type of residence* (rural or urban), and *mother’s age*. These variables were obtained directly from the Demographic and Health Surveys (DHS) datasets. The datasets from the 2010–11 and 2015 surveys were appended to create a pooled dataset. A new variable, *Year*, was generated to distinguish between the survey years.

### 2.3 Variables and definitions

#### 2.3.1 Stunting, wasting, and underweight

These variables were re-coded from the original data, and cases or variables with missing values were removed if they were few and did not significantly impact the analysis. Stunting, wasting, and underweight are critical indicators of child malnutrition and were defined using standard deviation scores (*z*-scores) based on the World Health Organization (WHO) growth standards (34):

- Stunting: Height-for-age *z*-score (HAZ) below −2 standard deviations.
- Wasting: Weight-for-height *z*-score (WHZ) below −2 standard deviations.
- Underweight: Weight-for-age *z*-score (WAZ) below −2 standard deviations.

#### 2.3.2 Double burden of malnutrition for children

The DBM for children was then defined as the presence of at least one of these undernutrition indicators combined with overweight (weight-for-height *Z*-score above +2 standard deviations). The percentage of DBM was calculated by summing the cases where both conditions were present.

#### 2.3.3 BMI categories for women

Body Mass Index (BMI) categories for women were defined as follows:

- Underweight: BMI ≤ 18.5
- Overweight: 24.9 < BMI ≤ 30
- Obesity: BMI > 30

The DBM was computed at the household level by identifying households where a woman was underweight (BMI ≤ 18.5) and simultaneously another woman was either overweight (BMI > 24.9 and ≤ 30) or obese (BMI > 30). We created household-level indicators for undernutrition (underweight) and overnutrition (overweight or obesity) and then defined DBM as the co-occurrence of these indicators within the same household.

### 2.4 Statistical analysis

#### 2.4.1 Descriptive statistics

Descriptive statistics were computed to summarise the demographic and socio-economic characteristics of the study population, stratified by survey year. This included summaries of continuous variables (e.g., age) and frequency distributions of categorical variables (e.g., SES, residence).

#### 2.4.2 Pooled logistic regression

Pooled logistic regression was employed in this study to analyse the association between various sociodemographic factors and the double burden of malnutrition (DBM) among women and children. This method allows combining data from different survey years (2010–2011 and 2015) to improve the statistical power and provide more robust estimates (35). The dependent variables were DBM among children and DBM at the household level for women. The independent variables included in the regression models were *Residence* (urban or rural), *Socio-economic Status (SES)* (wealth index), *Mother’s Education* (no education, primary, secondary, higher), *Mother’s Age*, *Child Age*, and *Year* (2010–2011, 2015). These variables were chosen based on their known influence on nutritional outcomes (36) through backward elimination. The logistic regression models were weighted using the DHS sample weights divided by 1,000,000 to account for the complex survey design of the DHS data, which involves stratification, clustering, and sampling weights. This adjustment ensured that the estimates are representative of the population and that the standard errors reflected the survey design.

The logistic regression models were specified as follows:

- For children:
$$\begin{array}{l} \mathrm{logistic}\;DBM_{\mathrm{children}}\;i.\mathrm{Residence}\;i.SES\;\mathrm{Mothers}:\mathrm{age}\;\\ \mathrm{Childage}\;i.\mathrm{Mothers}:edu\;i.\mathrm{Year}\;\left[\mathrm{pweight}=\mathrm{weightchi}\right] \end{array}$$
- For women:
$$\begin{array}{l} \mathrm{logistic}\;\mathrm{household}:DBM_{wom}\;i.\mathrm{Residence}\;i.SES\;\\ \mathrm{Mothers}:\mathrm{age}\;i.\mathrm{Mothers}:edu\;i.\mathrm{Year}\;\left[\mathrm{pweight}=\mathrm{weightchi}\right] \end{array}$$

Where:

- *DBMchildren* indicates the double burden of malnutrition among children.
- *household_DBMwom* indicates the double burden of malnutrition at the household level among women.
- *i*. denotes categorical variables.
- pweight = *weightchi* and pweight = *weightwom* are the survey weights for children and women, respectively.

#### 2.4.3 Oaxaca-Blinder decomposition

The Oaxaca-Blinder decomposition method was utilised in this study to explore the differences in the double burden of malnutrition (DBM) between the survey years 2010–2011 and 2015 among women and children in Zimbabwe. This technique decomposes the observed differences in DBM into two main components: the portion attributable to differences in the levels of explanatory variables (endowments) and the portion attributable to differences in the coefficients (effects of the explanatory variables) between the two survey years (37). This decomposition helps to identify whether changes in malnutrition outcomes are due to shifts in population characteristics or changes in how these characteristics influence malnutrition. The decomposition was performed separately for children and women, with DBM as the outcome variable.

##### 2.4.3.1 Specification of the Oaxaca-Blinder decomposition model

The independent variables included in the decomposition models were: *Residence* (urban or rural), *Socio-economic Status* (*S*ES), *Mother’s Education* (no education, primary, secondary, higher)*, Mother’s Age, Child Age* (for the children’s model) *and Year* (2010–2011 and 2015). The Oaxaca-Blinder decomposition was specified as follows:

- For children:
$$\begin{array}{l} \mathrm{oaxaca}\;DBM_{\mathrm{children}}\;\mathrm{Residence}\;SES\;\mathrm{Mothers}:\mathrm{age}\;\\ \mathrm{Childage}\;\mathrm{Mothers}:edu,by\;\left(\mathrm{Year}\right) \end{array}$$
- For women:
$$\begin{array}{l} \mathrm{oaxaca}\;\mathrm{household}:DBM_{wom}\;\mathrm{Residence}\;SES\;\\ \mathrm{Mothers}:\mathrm{age}\;\mathrm{Mothers}:edu,by\;\left(\mathrm{Year}\right) \end{array}$$

Where:

- *DBMchildren* indicate the double burden of malnutrition among children.
- *household_DBMwom* indicates the double burden of malnutrition at the household level among women.
- by (*Year*) specifies that the decomposition is performed by survey year.

The decomposition breaks down the differences in the mean outcomes between the two survey years into:

Endowments effect: This component measures the extent to which differences in the average levels of explanatory variables (e.g., changes in education levels, socio-economic status, or age distribution) contribute to the differences in DBM. It answers the question of how much of the change in DBM is due to differences in population characteristics between the 2 years.
Coefficients effect: This component captures the differences in the relationships (coefficients) between the explanatory variables and DBM across the survey years. It indicates how much of the change in DBM is due to changes in the impact of these characteristics on malnutrition. This effect can be interpreted as changes in the returns to characteristics.
Interaction effect: This additional component accounts for the simultaneous changes in endowments and coefficients.

All analyses were performed using Stata 17.0 (StataCorp LP, College Station, Texas, United States).

## 3 Results

Our study findings show that the average age of mothers in our sample was approximately 31 years, with ages ranging from 15 to 49 years, indicating a diverse age group (Table 1). Children’s ages average around 32 months, ranging from newborns to nearly 5 years old (Table 1). From 2010 to 2015, we observed a slight increase in the mean age of mothers from 30.73 years to 31.23 years, alongside a decrease in age variability (Table 1). There was a notable socio-economic improvement, with a decrease in the percentage of mothers in the poorest quartile from 20 to 16% and an increase in the richest quartile from 22 to 23% (Table 2). This shift is complemented by an increasing trend towards urban living, with the proportion of urban dwellers rising from 36 to 43% (Table 2). Similar trends are observed in the demographic characteristics of children (Table 2). The mean age of children increased slightly (Table 1), and there was a decrease in children from the poorest households alongside an increase in those from wealthier backgrounds (Table 2). The trend towards urbanisation is also mirrored in children’s data, with an increase in urban dwellers from 31 to 38% (Table 2).

**Table 1:** Descriptive statistics and overall household double burden of malnutrition (DBM) among women and children.

*Descriptive statistics of age characteristics for women and children*				
Variable	Mean	Std. dev.	Min	Max
Mother’s age (Years)	30.99	8.75	15	49
Child age (Months)	32.09	16.78	0	59
***Double Burden of Malnutrition (DBM) among women and children in 2010 and 2015***				
**Year**	**Overall Household DBM for Women**	**Overall DBM for Children**		
	***N*** **(%)**	***N* (%)**		
2010	2,526 (34.20)	534 (12.22)		
2015	2,526 (31.71)	649 (13.60)		

**Table 2:** Demographic characteristics for women and children.

*Demographic characteristics for women*							
Year	Total sample	Mean Age	SD	Socio-economic status		Residence status	
				Q1_poorest_	Q5_richest_	Rural	Urban
				*N* (%)	*N* (%)	*N* (%)	*N* (%)
2010	7,387	30.73	8.85	1,476 (19.98)	1,628 (22.04)	4,712 (63.79)	2,675 (36.21)
2015	7,965	31.23	8.66	1,322 (16.60)	2,069 (25.98)	4,508 (56.60)	3,457 (43.40)
*Demographic characteristics for children*							
2010	4,369	30.10	16.85	1,039 (23.78)	744 (17.03)	3,033 (69.42)	1,336 (30.58)
2015	4,778	33.14	16.65	951 (19.90)	1,017 (21.29)	2,962 (61.99)	1,816 (38.01)

Our study findings reveal a slight decrease in the overall household double burden of malnutrition (DBM) among women from 34% in 2010 to 32% in 2015 (Table 3). Conversely, the DBM among children increased from 12 to 14% during the same period (Table 3). Regarding socio-economic status, the prevalence of household DBM among women in 2010 was relatively uniform across different groups, with the highest recorded in the poorest quintile at 36% and the lowest in the richer quintile at 33% (Table 3). By 2015, there was a decline in household DBM across all socio-economic groups, with a notable reduction in the richest quintile to 29% (Table 3). The urban–rural divide in 2010 showed negligible differences in household DBM rates among women; however, by 2015, urban areas experienced a significant reduction in household DBM prevalence to 30% (Table 3). The distribution of DBM among children in 2010 varied widely by socio-economic status, from a low of 8% in the middle quintile to a high of 20% in the richest quintile (Table 3). The rural–urban divide in 2010 was stark, with urban areas experiencing higher rates of DBM at 19% compared to rural areas at 9% (Table 3). By 2015, there was an alarming increase in DBM among children in rural areas, which surged to 32%, while urban areas also saw a significant rise to 68% (Table 3).

**Table 3:** Prevalence of household double burden of malnutrition (DBM) among women and children by socio-economic status (SES) and residence.

*Among women in 2010 and 2015*							
Year	Socio-economic status					Residence status	
	Q1_poorest_	Q2_poorer_	Q3_middle_	Q4_rich_	Q5_richest_	Rural	Urban
	*N* (%)	*N* (%)	*N* (%)	*N* (%)	*N* (%)	*N* (%)	*N* (%)
2010	532 (36.04)	425 (32.87)	427 (32.77)	607 (35.98)	535 (32.86)	1,614 (34.25)	912 (34.09)
2015	464 (35.10)	402 (32.87)	420 (33.55)	639 (30.44)	601 (29.05)	1,514 (33.58)	1,012 (29.27)
*Among children in 2010 and 2015*							
2010	100 (9.62)	78 (9.11)	65 (8.21)	141 (15.03)	150 (20.16)	275 (9.07)	259 (19.39)
2015	84 (3.58)	127 (5.37)	59 (2.49)	150 (6.29)	229 (9.64)	207 (31.90)	442 (68.10)

Our pooled logistic regression analysis provided several insights into the factors associated with the Double Burden of Malnutrition (DBM) among children and women. The effect of the mother’s age on DBM among children was statistically insignificant, with an odds ratio (OR) of 0.9997 (95% CI: [0.99, 1.01], *p* = 0.97), indicating that the mother’s age does not significantly influence the likelihood of DBM in children (Table 4). The residence status revealed that children in rural areas had significantly higher odds (OR = 2.43; 95% CI: [1.73, 3.43], *p* < 0.001) of experiencing DBM compared to their urban counterparts. Socioeconomic status (SES) did not show a significant effect on DBM among children, with odds ratios close to 1 and non-significant *p* values for all SES categories (Table 4). Similarly, the mother’s education level showed no statistically significant association with DBM, with odds ratios slightly above or below 1 and non-significant *p*-values. A significant temporal increase in the odds of DBM from 2010 to 2015 was observed, with an OR of 2.60 (95% CI: [2.14, 3.16], *p* < 0.00), indicating a substantial rise in the risk of DBM over the 5-year period (Table 4).

**Table 4:** Pooled logistic regression of double burden of malnutrition among children.

	Children			Women		
Variable	Odds ratio	*p* value	95% confidence interval	Odds ratio	*p* value	95% confidence interval
Mother’s age						
	0.9997	0.97	[0.99, 1.01]	1.00	0.631	[0.99, 1.01]
Child age						
	1.01	0.06	[1.00, 1.01]			
Residence						
Urban	Reference	Reference	Reference	Reference	Reference	Reference
Rural	2.43	0.00	[1.73, 3.43]	1.09	0.357	[0.90, 1.33]
Socio-economic status (SES)						
Poorest	Reference	Reference	Reference	Reference	Reference	Reference
Poorer	0.83	0.23	[0.61, 1.12]	1.02	0.782	[0.88, 1.19]
Middle	1.24	0.24	[0.87, 1.77]	1.05	0.547	[0.89, 1.24]
Richer	1.01	0.96	[0.69, 1.48]	1.09	0.374	[0.90, 1.34]
Richest	0.76	0.21	[0.49, 1.17]	1.01	0.950	[0.79, 1.29]
Mother’s education						
No education	Reference	Reference	Reference	Reference	Reference	Reference
Primary Education	1.13	0.74	[0.55, 2.31]	0.92	0.69	[0.62, 1.37]
Secondary Education	1.35	0.41	[0.66, 2.75]	0.92	0.67	[0.62, 1.37]
Higher Education	0.87	0.74	[0.39, 1.95]	0.89	0.61	[0.55, 1.42]
Year						
2010	Reference	Reference	Reference	Reference	Reference	Reference
2015	2.60	0.00	[2.14, 3.16]	1.49	0.00	[1.35, 1.65]
Constant						
	3.13	0.01	[1.27, 7.73]	0.54	0.01	[0.33, 0.88]

For women, the analysis showed that the mother’s age had no significant impact on the likelihood of experiencing household DBM, with an OR of 1.00 (95% CI: [0.99, 1.01], *p* = 0.631). While women in rural areas had slightly higher odds of experiencing household DBM (OR = 1.09; 95% CI: [0.90, 1.33], *p* = 0.357), this difference was not statistically significant (Table 4). SES similarly had no significant effect, with odds ratios near 1 across all socioeconomic groups and non-significant p-values. The education level of mothers also showed no significant association with household DBM, with all educational categories yielding odds ratios close to 1 and non-significant *p* values. A critical finding was the significant increase in the odds of household DBM among women from 2010 to 2015, with an OR of 1.49 (95% CI: [1.35, 1.65], *p* < 0.001), highlighting a concerning rise in DBM risk over time (Table 4). The model’s constant for children had an OR of 3.13 (95% CI: [1.27, 7.73], *p* = 0.01), indicating that the baseline odds of DBM, when all other variables are at their reference levels, are significantly above 1, whereas the constant for women had an OR of 0.54 (95% CI: [0.33, 0.88], *p* = 0.01), indicating significantly lower baseline odds (Table 4).

The Oaxaca-Blinder decomposition analysis of the Double Burden of Malnutrition (DBM) among children, as presented in Table 5, reveals significant insights into the changes observed between 2010 and 2015. In 2010, the coefficient for DBM was 0.88 (95% CI: [0.87, 0.89], *p* < 0.001), which increased to 0.95 in 2015 (95% CI: [0.94, 0.95], *p* < 0.001), indicating a statistically significant rise in DBM. The overall difference between these years was −0.06 (95% CI: [−0.07, −0.05], *p* < 0.001), highlighting a meaningful increase in the prevalence of DBM among children over this period.

**Table 5:** Oaxaca-Blinder decomposition of double burden of malnutrition among children and women.

	Children				Women			
Variable	Coefficient	Contribution %	*p* value	95% confidence interval	Coefficient	Contribution %	*p* value	95% confidence interval
Overall								
2010	0.88		0.00	[0.87, 0.89]	0.33		0.00	[0.32, 0.35]
2015	0.95		0.00	[0.94, 0.95]	0.43		0.00	[0.42, 0.45]
Difference	−0.06		0.00	[−0.07, −0.05]	−0.10		0.00	[−0.12, −0.08]
Endowments	0.00	7.44	0.00	[0.00, 0.01]	0.00	2.39	0.17	[−0.00, 0.01]
Coefficients	−0.07	185.35	0.00	[−0.08, −0.05]	−0.10	−29.37	0.00	[−0.12, −0.08]
Interaction	0.00	3.25	0.27	[−0.00, 0.01]	−0.00	−1.41	0.58	[−0.01, 0.00]
Endowments								
Residence	0.00	4.88	0.00	[0.00, 0.00]	0.00	1.83	0.35	[−0.00, 0.01]
Socio-economic status	0.00	1.14	0.46	[−0.00, 0.00]	−0.00	−1.13	0.59	[−0.01, 0.00]
Mothers age	0.00	0.66	0.34	[−0.00, 0.00]	0.00	0.67	0.47	[−0.00, 0.00]
Child age	0.00	-	0.70	[−0.00, 0.00]				
Mother education	0.00	0.44	0.67	[−0.00, 0.00]	0.00	1.02	0.46	[−0.00, 0.00]
Coefficients								
Residence	0.08	130.84	0.01	[0.02, 0.14]	−0.05	−53.05	0.34	[−0.16, 0.05]
Socio-economic status	−0.01	−18.43	0.58	[−0.05, 0.03]	−0.04	−40.52	0.28	[−0.11, 0.03]
Mothers age	0.01	11.98	0.78	[−0.04, 0.06]	0.06	58.82	0.20	[−0.03, 0.15]
Child age	0.02	-	0.04	[0.00, 0.05]				
Mother education	0.01	20.98	0.55	[−0.03, 0.05]	0.01	5.38	0.89	[−0.07, 0.08]
Constant	−0.18		0.00	[−0.29, −0.07]	−0.07		0.47	[−0.26, 0.12]
Interaction								
Residence	0.00	6.26	0.02	[0.00, 0.01]	−0.00	−2.54	0.34	[−0.01, 0.00]
Socio-economic status	0.00	1.38	0.58	[−0.00, 0.00]	0.00	3.03	0.28	[−0.00, 0.01]
Mothers age	0.00	−0.33	0.78	[−0.00, 0.00]	−0.00	−1.63	0.21	[−0.00, 0.00]
Child age	−0.00	-	0.05	[−0.00, 0.00]				
Mother education	−0.00	−1.07	0.55	[−0.00, 0.00]	−0.00	−0.27	0.89	[−0.00, 0.00]

The decomposition analysis showed that changes in the characteristics of the population (endowments) contributed minimally to this change, with a coefficient of 0.00 (95% CI: [0.00, 0.01], *p* < 0.001) and accounting for 7.44% of the difference (Table 5). In contrast, changes in the effects of these characteristics (coefficients) had a substantial negative contribution of −0.07 (95% CI: [−0.08, −0.05], *p* < 0.001), contributing 185.35% to the increase in DBM. Interaction effects were minimal and statistically insignificant, with a coefficient of 0.00 (95% CI: [−0.00, 0.01], *p* = 0.27), contributing 3.25% to the overall change. When examining specific endowments, residence was the only factor that showed a statistically significant contribution (coefficient of 0.00, 95% CI: [0.00, 0.00], *p* < 0.001), contributing 4.88% to the change. Socio-economic status, mother’s age, child’s age, and mother’s education had negligible and non-significant contributions, each with coefficients close to 0 and *p* values exceeding 0.05 (Table 5). This suggests that changes in residence had a small but significant impact on DBM.

For the coefficients, residence had a significant positive contribution of 0.08 (95% CI: [0.02, 0.14], *p* = 0.01), indicating that changes in the effect of residence on DBM were significant (Table 5). Socioeconomic status had a negative but non-significant contribution of −0.01 (95% CI: [−0.05, 0.03], *p* = 0.58). The mother’s age had a non-significant positive contribution of 0.01 (95% CI: [−0.04, 0.06], *p* = 0.78), while the child’s age had a small but significant positive contribution of 0.02 (95% CI: [0.00, 0.05], *p* = 0.04). Mother’s education had a non-significant positive contribution of 0.01 (95% CI: [−0.03, 0.05], *p* = 0.55). The constant term showed a significant negative contribution of −0.18 (95% CI: [−0.29, −0.07], *p* < 0.001), indicating a baseline decrease when all other variables are held constant. Regarding interaction effects, residence had a small but significant positive interaction effect of 0.00 (95% CI: [0.00, 0.01], *p* = 0.02). Socio-economic status, mother’s age, child’s age, and mother’s education had negligible and statistically non-significant interaction effects, with coefficients close to 0 and *p* values exceeding 0.05 (Table 5).

For women, the Oaxaca-Blinder decomposition analysis of Household DBM between 2010 and 2015 also reveals significant changes (Table 5). In 2010, the coefficient for DBM was 0.33 (95% CI: [0.32, 0.35], *p* < 0.001), which increased to 0.43 in 2015 (95% CI: [0.42, 0.45], *p* < 0.001), indicating a statistically significant rise in DBM (Table 5). The overall difference of −0.10 (95% CI: [−0.12, −0.08], *p* < 0.001) suggests a meaningful increase in DBM among women during this period. Endowments contributed minimally to this increase, with a coefficient of 0.00 (95% CI: [−0.00, 0.01], *p* = 0.17), accounting for only 2.39% of the difference. In contrast, the coefficients component had a significant negative contribution of −0.10 (95% CI: [−0.12, −0.08], *p* < 0.001), contributing −29.37% to the increase in DBM. Interaction effects were minimal and statistically insignificant, with a coefficient of −0.00 (95% CI: [−0.01, 0.00], *p* = 0.58), contributing −1.41% (Table 5).

Specific endowments showed varied contributions, but none were statistically significant. Residence had a small, non-significant positive contribution (coefficient of 0.00, 95% CI: [−0.00, 0.01], *p* = 0.35), contributing 1.83% to the difference (Table 5). Socio-economic status, mother’s age, and mother’s education also had non-significant contributions. For the coefficients, residence had a non-significant negative contribution of −0.05 (95% CI: [−0.16, 0.05], *p* = 0.34), while socio-economic status contributed −0.04 (95% CI: [−0.11, 0.03], *p* = 0.28). The mother’s age had a positive but non-significant contribution (coefficient of 0.06, 95% CI: [−0.03, 0.15], *p* = 0.20), and mother’s education had a minimal, non-significant contribution (coefficient of 0.01, 95% CI: [−0.07, 0.08], *p* = 0.89). The constant term showed a non-significant negative contribution (coefficient of −0.07, 95% CI: [−0.26, 0.12], *p* = 0.47).

## 4 Discussion

The prevalence of DBM among women and children is on an upward trajectory in Zimbabwe. Through a pooled logistic regression analysis, this study offers valuable insight into the factors associated with DBM using nationally representative datasets. Additionally, using the Oaxaca-Blinder decomposition technique, this study investigated whether the change in DBM over time among women and children was due to shifts in population characteristics (composition/endowment effects) or changes in how these characteristics influence malnutrition (coefficient/behavioural effects). The socio-economic disparities in DBM are also noteworthy. The reduction in DBM among women in the richest quintile suggests that wealthier households are better able to mitigate the risks associated with DBM, likely due to better access to diverse and quality foods, healthcare, and information about healthy lifestyles (14, 38–41). Conversely, the persistence of DBM across poorer socio-economic groups underscores the need for targeted interventions that address ends of the malnutrition spectrum—undernutrition and overweight/obesity.

The regression analyses showed a significant temporal increase (from 2010 to 2015) in the odds of DBM among women and children, indicating a concerning rise in the risk of DBM in households over the 5 years. This increased risk can be attributed to the changes happening in the continent: increased economic development and rapid urbanisation (42), reduced physical activity and increased intake of processed foods high in sugars, salts and fats (westernised diet) (43–46). Moreover, in as much as nutritional transition is a global phenomenon, studies have shown that changes in dietary patterns and increasing sedentary lifestyles in some low-income settings like Zimbabwe are occurring more rapidly than in high-income countries (11, 47–56).

Our findings also suggested that children in rural areas had higher odds of experiencing DBM compared to children in urban areas. This is corroborated by other studies which reported that children in rural areas are likely to be food insecure and malnourished (57–60). The higher prevalence of DBM in rural areas can be linked to limited access to diverse and nutritious foods (1, 14, 31), inadequate healthcare services (1, 61, 62), and lower socio-economic status (7, 17, 63, 64), which collectively contribute to the increased vulnerability of these populations.

The Oaxaca-Blinder decomposition demonstrated a considerable increase (6%) in DBM between 2010/11 and 2015, with demographic and socio-economic factors explaining 7.44% of the total change in the rates of DBM among children. Of this 7.44%, approximately 5% was due to changes in residence status over the two time periods. This can be partly explained by the increase in the rates of DBM in rural (30%) and urban areas (30%) over the two time periods, although higher levels of DBM were observed in urban areas over the two periods. These higher levels of DBM in urban areas can be explained by research demonstrating that food markers in these areas are replacing fresh foods with processed foods, often commercially prepared (65–68). As explained above, the rise of DBM in rural areas is established in other research showing that children in rural areas have a higher likelihood of being food insecure and experiencing malnutrition (57, 58). Therefore, to address DBM, stakeholders need to tailor solutions to the specific contexts of rural and urban settings. The implications of these findings are significant, as they highlight the need for targeted public health interventions that address undernutrition and overweight/obesity. Policymakers and health practitioners must consider rural communities’ unique challenges, such as food insecurity and limited healthcare access while addressing the broader impacts of economic development and urbanisation. Interventions should promote physical activity, improve access to healthy foods, and implement education campaigns to encourage healthier dietary choices. Addressing DBM requires a multifaceted approach considering the complex interplay of socio-economic, environmental, and behavioural factors contributing to this growing public health challenge.

The decomposition analysis also shows that most of the variation in the extent of DBM among children between 2010/11 and 2015 is due to changes in the coefficient. The change in the coefficient of residence contributed the most (+130.84%) towards increasing the probability of DBM among children over the two time periods. This conclusively demonstrates that the worsening DBM among children is primarily driven by changes in how residence status impacts nutritional outcomes rather than the shifts in the distribution of residence status over the two periods. Existing literature supports this, highlighting how urbanisation and rural–urban migration influence dietary patterns and physical activity levels, thereby affecting nutritional health (69–72). Studies have shown that urban areas, while offering better access to healthcare and diversified food options, often expose residents to unhealthy dietary practises and sedentary lifestyles, contributing to rising obesity rates (73–79).

Conversely, rural areas, despite being traditionally associated with undernutrition, are now experiencing the dual challenges of food insecurity and increasing prevalence of overweight/obesity due to limited access to healthy foods and healthcare services (80–82). This dual burden is exacerbated by economic transitions affecting food systems and rural lifestyle choices. This underscores the need for focused interventions that specifically address how evolving residence status conditions influence children’s nutritional health, aiming to mitigate the increasing burden of malnutrition in households.

For women, the coefficient effect accounted for most (−29.37%) of the unexplained differences in DBM between 2010/11 and 2015. Since the contributions of the change in coefficients for residence status, mother’s age, mother’s education and SES were not statistically significant, other factors outside this model were responsible for the narrowing effect of this coefficient. Our study has ably pointed out that DBM among women and children in Zimbabwe is on an uptick, and residence has come out strongly as a driver of this increase among children. This is a reminder for Zimbabwe to consolidate and fast-track its efforts to address DBM in line with the World Health Organization’s (WHO) recommended Double Duty Actions (DDA) (83). The DDA framework focuses on promoting and protecting exclusive breastfeeding in the first 6 months, promoting appropriate early and complementary feeding in infants, maternal nutrition and antenatal care programmes, regulations on food marketing, and school food policies and programmes (84). Regarding the effect of residence, our research points out the need to pay attention to the role of urban–rural inequalities and specific contexts in urban and rural areas in facilitating increases in DBM among women and children.

The main strength of this study lies in its use of two nationally representative datasets to explain changes in the Double Burden of Malnutrition (DBM) among children and women. By leveraging large, comprehensive datasets, the study ensures that the findings are generalisable and reflective of the broader population, enhancing the reliability and validity of the results. Additionally, using the Oaxaca-Blinder decomposition technique is a significant methodological strength. This technique allowed for a detailed examination of the sources of group differences, providing valuable insights into the specific factors contributing to changes in DBM over time. Such insights are crucial for policymakers and researchers as they offer a nuanced understanding of the mechanisms driving these differences, informing more targeted and effective interventions.

However, this study is not without limitations. Firstly, the cross-sectional study design limits the ability to establish causal-effect relationships. While the analysis can identify associations and trends, it cannot definitively determine whether specific factors cause observed changes in DBM. Longitudinal studies would be necessary to establish causality and better understand the temporal dynamics of DBM. Secondly, the study measured the socio-economic status (SES) of households using the wealth index, which is based on household assets and standard of living. While the wealth index is a widely used proxy for SES, it may not capture all dimensions of socio-economic status. The contribution of SES to DBM might have been different if other measures, such as household income, expenditures, or consumption, were used as proxies. These alternative measures can provide a more dynamic and direct reflection of a household’s economic situation. For instance, household income captures earnings and financial resources more directly, expenditures reflect spending behaviour, and consumption measures the actual use of goods and services. Each of these proxies can yield different insights into the relationship between SES and DBM, potentially leading to different conclusions about the impact of socioeconomic factors.

Therefore, the results of this study must be interpreted given these limitations. Future research could benefit from using longitudinal designs to explore causal pathways and employing various measures of SES to capture a more comprehensive picture of the economic factors influencing DBM. Despite these limitations, the study’s strengths in data representativeness and methodological rigour provide valuable contributions to understanding DBM and its determinants.

## 5 Conclusion

This study highlights the growing Double Burden of Malnutrition (DBM) among women and children in Zimbabwe, driven significantly by changes in how residence status affects nutritional outcomes. The regression and decomposition analyses underscore that residence status, particularly in rural areas, is a critical factor influencing DBM, highlighting the need for targeted interventions to address urban–rural disparities. While wealthier households show better resilience against DBM, poorer groups remain vulnerable, necessitating comprehensive strategies that address undernutrition and overweight/obesity. Despite the limitations of a cross-sectional design and the use of the wealth index, the study provides valuable insights through nationally representative data and advanced analytical techniques. Future research should adopt longitudinal approaches and explore diverse socio-economic measures to deepen understanding. To mitigate the rising DBM, context-specific public health strategies are essential, aligning with WHO’s Double Duty Actions to improve the nutritional health of Zimbabwe’s population.

## Data Availability

Publicly available datasets were analysed in this study. This data can be found here: all data sets are publicly available on the Demographic Health Survey website at: https://dhsprogram.com/what-we-do/survey/survey-display-406.cfm.
